# Allergic Reactions Including Anaphylaxis After Receipt of the First Dose of Moderna COVID-19 Vaccine — United States, December 21, 2020–January 10, 2021

**DOI:** 10.15585/mmwr.mm7004e1

**Published:** 2021-01-29

**Authors:** CDC COVID-19 Response Team, Food and Drug Administration

As of January 20, 2021, a total of 24,135,690 cases of coronavirus disease 2019 (COVID-19) and 400,306 associated deaths had been reported in the United States (https://covid.cdc.gov/covid-data-tracker/#cases_casesper100klast7days). On December 18, 2020, the Food and Drug Administration (FDA) issued an Emergency Use Authorization (EUA) for Moderna COVID-19 vaccine administered as 2 doses, 1 month apart to prevent COVID-19. On December 19, 2020, the Advisory Committee on Immunization Practices (ACIP) issued an interim recommendation for use of Moderna COVID-19 vaccine (*1*). As of January 10, 2021, a reported 4,041,396 first doses of Moderna COVID-19 vaccine had been administered in the United States, and reports of 1,266 (0.03%) adverse events after receipt of Moderna COVID-19 vaccine were submitted to the Vaccine Adverse Event Reporting System (VAERS). Among these, 108 case reports were identified for further review as possible cases of severe allergic reaction, including anaphylaxis. Anaphylaxis is a life-threatening allergic reaction that occurs rarely after vaccination, with onset typically within minutes to hours (*2*). Among these case reports, 10 cases were determined to be anaphylaxis (a rate of 2.5 anaphylaxis cases per million Moderna COVID-19 vaccine doses administered), including nine in persons with a documented history of allergies or allergic reactions, five of whom had a previous history of anaphylaxis. The median interval from vaccine receipt to symptom onset was 7.5 minutes (range = 1–45 minutes). Among eight persons with follow-up information available, all had recovered or been discharged home. Among the remaining case reports that were determined not to be anaphylaxis, 47 were assessed to be nonanaphylaxis allergic reactions, and 47 were considered nonallergic adverse events. For four case reports, investigators have been unable to obtain sufficient information to assess the likelihood of anaphylaxis. This report summarizes the clinical and epidemiologic characteristics of case reports of allergic reactions, including anaphylaxis and nonanaphylaxis allergic reactions, after receipt of the first dose of Moderna COVID-19 vaccine during December 21, 2020–January 10, 2021, in the United States. CDC has issued updated interim clinical considerations for use of mRNA COVID-19 vaccines currently authorized in the United States (*3*) and interim considerations for preparing for the potential management of anaphylaxis (*4*).

Using methods previously described (*5*), CDC and FDA identified reports of suspected anaphylaxis in VAERS, the national passive surveillance (i.e., spontaneous reporting) system for monitoring adverse events after immunization (*6*). CDC physicians screened VAERS reports describing suspected severe allergic reactions and anaphylaxis and applied Brighton Collaboration case definition criteria for anaphylaxis* (*7*). After initial screening, reports with sufficient evidence to suggest anaphylaxis were followed up by collecting information from medical records and through direct outreach to health care facilities and treating health care providers, and, in some cases, vaccine recipients. Physician reviewers classified all initially identified case reports as anaphylaxis or not anaphylaxis and used clinical judgment to further categorize reports that were considered not anaphylaxis as nonanaphylaxis allergic reactions or nonallergic adverse events. Nonallergic adverse events, mostly vasovagal (e.g., fainting or the sensation of fainting) or suspected anxiety-related, were excluded from the final analyses. Anaphylaxis and nonanaphylaxis allergic reaction cases with symptom onset occurring later than the day after vaccination (i.e., outside the 0–1-day risk window) were also excluded because of the difficulty in clearly attributing allergic reactions with onset later than this to vaccination.^†^

During December 21, 2020–January 10, 2021, the administration of 4,041,396 first doses of Moderna COVID-19 vaccine (2,465,411 to females [61%], 1,450,966 to males [36%], and 125,019 to persons whose sex was not recorded [3%]) was reported to CDC. During the same period, reports of 1,266 (0.03%) adverse events after receipt of the first dose of Moderna COVID-19 vaccine had been submitted to VAERS. Among these, 108 case reports were identified for further review as possible cases of severe allergic reaction, including anaphylaxis, based on descriptions of signs and symptoms; 10 of these reports, all describing events in females, met the Brighton Collaboration case definition criteria for anaphylaxis (Table 1), corresponding to an initial estimated rate of 2.5 anaphylaxis cases per million first Moderna COVID-19 vaccine doses administered. The median age of persons with anaphylaxis was 47 years (range = 31–63 years). The median interval from vaccine receipt to symptom onset was 7.5 minutes (range = 1–45 minutes); nine patients had onset within 15 minutes, and one had onset after 30 minutes (Figure). In all 10 reports, patients received epinephrine as part of initial emergency treatment; the route of administration was confirmed or presumed to be intramuscular based on the description of treatment and the clinical course of the event as documented in the VAERS report. Six patients were hospitalized (including five in intensive care, four of whom required endotracheal intubation), and four were treated in an emergency department; eight patients with follow-up information available are known to have been discharged home or had recovered at the time of report to VAERS. No deaths from anaphylaxis were reported after receipt of Moderna COVID-19 vaccine. Nine of the 10 anaphylaxis case reports included a patient history of allergies or allergic reactions, including to drugs (six), contrast media (two), and foods (one); five patients had experienced an episode of anaphylaxis in the past, none of which was associated with receipt of a vaccine (Table 2). No geographic clustering of anaphylaxis cases was observed, and the cases occurred after receipt of doses from multiple vaccine lots. At the time of this publication, despite follow-up efforts, investigators have been unable to obtain sufficient information to assess the likelihood of anaphylaxis in four of the initial 108 suspected cases reported.

**TABLE 1:** Characteristics of reported cases of anaphylaxis (n = 10) after receipt of the first dose of Moderna COVID-19 vaccine — Vaccine Adverse Events Reporting System (VAERS), United States, December 21, 2020–January 10, 2021

Age, yrs	Sex	Past history		Onset after receipt (mins)	Signs and symptoms	Treatment setting^†^	Epi received	Brighton level^§^	Outcome or disposition^¶^
		Allergies or allergic reactions*	Previous anaphylaxis episode						
37	F	Penicillin, phenytoin, ibuprofen	No	1	Respiratory failure, vomiting	Inpatient	Yes	2	Discharged home
39	F	Penicillin, aloe	Yes, penicillin	2	Decreased peripheral perfusion, persistent dry cough, nausea	Inpatient	Yes	3	Discharged home
63	F	Acetaminophen, azithromycin	No	4	Periorbital edema, nausea	ED	Yes	2	Not specified
55	F	Multiple unspecified environmental and food allergies	Yes, unspecified	5	Hypotension, wheezing	Inpatient	Yes	2	Not specified
31	F	No	No	5	Diffuse erythematous rash, throat swelling	ED	Yes	1	Discharged home
49	F	Gadolinium, iodine	Yes, gadolinium, iodine	10	Diffuse erythematous rash, tongue swelling, wheezing	ED	Yes	1	Recovered at time of report
37	F	Unspecified intravenous contrast dye, penicillin	Yes, intravenous contrast dye	11	Generalized urticarial rash, tongue swelling	Inpatient	Yes	1	Discharged home
50	F	Unspecified allergies or allergic reactions	Yes, unspecified	12	Diffuse erythematous rash, wheezing	Inpatient	Yes	1	Discharged home
57	F	Multiple drugs including penicillin and sulfa	No	13	Periorbital edema, tongue swelling	ED	Yes	1	Recovered at time of report
44	F	Morphine, codeine	No	45	Diffuse erythematous rash, marked tongue swelling	Inpatient	Yes	1	Discharged home

**Abbreviations**: COVID-19 = coronavirus disease 2019; ED = emergency department; Epi = epinephrine; F = female.

* As documented in the VAERS report or medical records, or through confirmation with the treating health care provider or the patients themselves.

^†^ Inpatient hospitalization.

^§^ The Brighton Collaboration case definition uses combinations of symptoms to define levels of diagnostic certainty. Brighton level 1 represents the highest level of diagnostic certainty that a reported case is indeed a case of anaphylaxis; levels 2 and 3 are successively lower levels of diagnostic certainty. Level 4 is a case reported as anaphylaxis but that does not meet the Brighton Collaboration case definition. Level 5 is a case that was neither reported as anaphylaxis nor meets the case definition (https://doi.org/10.1016/j.vaccine.2007.02.064).

^¶^ As documented in the description of the adverse event in the VAERS report in Box 18 or as documented in recovery status in Box 20.

**FIGURE:**
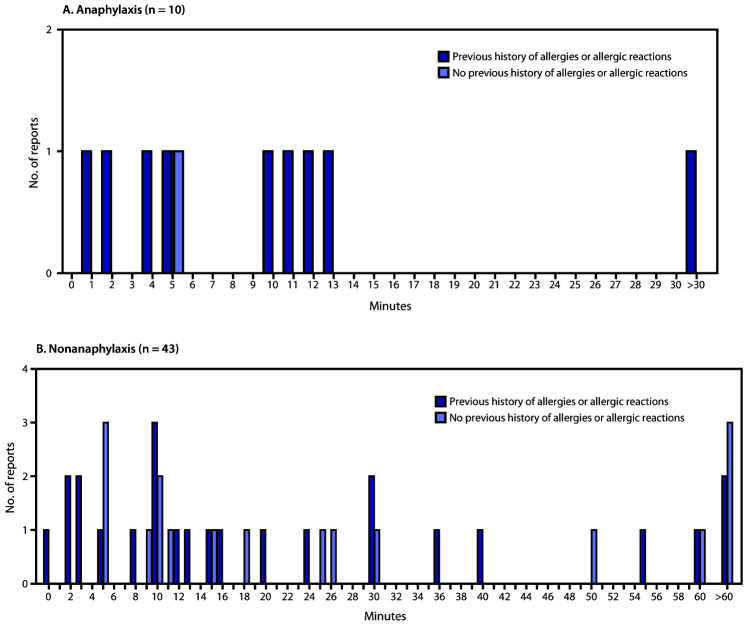
Minutes from vaccine receipt to onset of anaphylaxis (A)* and nonanaphylaxis allergic reactions (B)^†^ after receipt of the first dose of Moderna COVID-19 vaccine — Vaccine Adverse Events Reporting System (VAERS), United States, December 21, 2020–January 10, 2021 **Abbreviation:** COVID-19 = coronavirus disease 2019. * The interval from vaccine receipt to symptom onset was >30 minutes for one anaphylaxis case (45 minutes). ^†^ The interval from vaccine receipt to symptom onset was ≥60 minutes for three nonanaphylaxis patients who had a documented history of allergies or allergic reactions at 60, 90, and 98 minutes and for four who did not have a documented history of allergies or allergic reactions (60 minutes, 10 hours, 20 hours, and 24 hours). The interval from vaccine receipt to symptom onset was missing in two case reports, both of which documented a history of allergies or allergic reactions. Four cases of nonanaphylaxis allergic reactions with symptom onset occurring later than the day after vaccination (i.e., outside of the 0–1-day risk window) were excluded from the final analysis.

**TABLE 2:** Characteristics of patients with reported anaphylaxis and nonanaphylaxis allergic reactions after receipt of the first dose of Moderna COVID-19 vaccine — Vaccine Adverse Events Reporting System (VAERS), United States, December 21, 2020–January 10, 2021

Characteristic	Type of reported reaction, no. (%)	
	Anaphylaxis (n = 10)	Nonanaphylaxis allergic reactions (n = 43)*
Median age, yrs (range)	47 (31–63)	43 (22–96)
Female	10 (100)	39 (91)
Minutes to symptom onset, median (range)	7.5 (1–45)	15 (<1–1,440 [24 hrs])
Symptom onset ≤15 mins	9 (90)	21 (51)^†^
Symptom onset ≤30 mins	9 (90)	30 (73)^†^
Documented history of allergies or allergic reactions	9 (90)^§^	26 (60)

**Abbreviation**: COVID-19 = coronavirus disease 2019.

* Four of the initial 47 nonanaphylaxis allergic reaction reports were excluded from the final analysis because symptom onset occurred later than the day after vaccination (i.e., outside the 0–1-day risk window).

^†^ Two nonanaphylaxis allergic reaction reports were missing information on time of symptom onset; percentage calculated among 41 case reports with onset documented.

^§^ Five anaphylaxis reports included a patient history of a previous anaphylaxis episode.

Among the 43 cases of nonanaphylaxis allergic reaction after receipt of Moderna COVID-19 vaccination with symptom onset within the 0–1-day risk window, 26 (60%) were classified as nonserious.^§^ Commonly reported symptoms included pruritus, rash, itchy sensations in the mouth and throat, sensations of throat closure, and respiratory symptoms. The median patient age was 43 years (range = 22–96 years), and 39 (91%) of the reported reactions occurred in women. The median interval from vaccine receipt to symptom onset was 15 minutes (range = <1 minute–24 hours); in 30 (73%) cases, onset occurred within 30 minutes, in 11 cases, onset occurred after 30 minutes, and for two cases, time of onset was missing. For 26 (60%) case reports, a past history of allergies or allergic reactions, mostly to foods and drugs, was documented (Figure).

## Discussion

Early safety monitoring of Moderna COVID-19 vaccine detected 10 cases of anaphylaxis after reported administration of 4,041,396 first doses of Moderna COVID-19 vaccine (2.5 cases per million Moderna COVID-19 vaccine doses administered) as well as cases of less severe nonanaphylaxis allergic reactions, based on U.S. data for December 21, 2020–January 10, 2021. Anaphylaxis is potentially life-threatening and requires immediate treatment (*4*). Based on this early monitoring, anaphylaxis after receipt of Moderna COVID-19 vaccine appears to be a rare event; however, comparisons of anaphylaxis risk with that associated with non–COVID-19 vaccines are constrained at this time by the limited data available this early in the COVID-19 vaccination program. A previous analysis of the Pfizer-BioNTech COVID-19 vaccine, also an mRNA vaccine, estimated an initial rate of 11.1 cases per million doses administered after receipt of the first dose of the Pfizer-BioNTech vaccine (*5*). CDC and FDA will continue enhanced monitoring for anaphylaxis among recipients of COVID-19 vaccines and will review case reports to VAERS.

In nine of 10 cases of anaphylaxis after receipt of Moderna COVID-19 vaccine, patients had symptom onset within 30 minutes of vaccination, and nine anaphylaxis patients also had a history of allergies or allergic reactions, including some with previous anaphylaxis events; up to 30% of persons in the general population might have some type of allergy or history of allergic reactions.^¶^ All 10 anaphylaxis cases reported after receipt of Moderna COVID-19 vaccine occurred in women. Whereas a previous review of anaphylaxis reports to VAERS found that 80% of cases reported in adults involved females (*8*), the current finding could be affected by the observation that more women than men had received a first dose of Moderna COVID-19 vaccine during the analytic period (61% of doses administered versus 36%, respectively). In a previous analysis of the Pfizer-BioNTech COVID-19 vaccine, two thirds of first doses were administered in women (*5*). The clinical and epidemiologic characteristics of anaphylaxis case reports after receipt of Moderna COVID-19 vaccine are similar to those reported after receipt of the Pfizer-BioNTech COVID-19 vaccine (*5*). For both vaccines, symptom onset after vaccination occurred quickly, usually within minutes. A strong female predominance of anaphylaxis case reports exists for both vaccines. Finally, many persons experiencing anaphylaxis after receiving either vaccine had a history of allergies or allergic reactions, with several having experienced an anaphylaxis episode in the past. Similar patient characteristics in case reports of nonanaphylaxis allergic reactions were observed among the two vaccines.

The findings in this report are subject to at least two limitations. First, analyses of passive surveillance data include reporting biases, both underreporting because of lack of awareness or compliance with reporting requirements and reporting guidance, as well as stimulated reporting related to increased awareness from media or other public information sources. Second, incomplete information in reports and potential data lags because of processing times might result in an undercount of cases, and lags in reporting for vaccine doses administered might underestimate denominator data. However, reporting efficiency to VAERS for clinically severe adverse events is believed to be high (*9*). It is reasonable to expect that diagnosis and reporting of an acute and clinically severe condition such as anaphylaxis occurs relatively quickly, and VAERS is likely sensitive at capturing anaphylaxis cases occurring after COVID-19 vaccination.

Mortality from COVID-19 in populations at increased risk for severe illness is substantial (*10*), and treatment options are limited. Widespread vaccination against COVID-19 with highly effective vaccines represents a critical tool in efforts to control the pandemic and save lives. CDC and FDA will continue to monitor for adverse events, including anaphylaxis, after administration of COVID-19 vaccines and will regularly assess the benefits and risks of vaccination in the context of the evolving epidemiology of the pandemic. Continued monitoring in VAERS and additional monitoring in population-based surveillance systems, such as the CDC’s Vaccine Safety Datalink (https://www.cdc.gov/vaccinesafety/ensuringsafety/monitoring/vsd/index.html), will help to further characterize the risk for anaphylaxis after administration of COVID-19 vaccines.

CDC guidance on use of mRNA COVID-19 vaccines and management of anaphylaxis is available (*3*,*4*). Persons with an immediate allergic reaction to the first dose of an mRNA COVID-19 vaccine should not receive additional doses of either of the mRNA COVID-19 vaccines. In addition to screening for contraindications and precautions before administering COVID-19 vaccines, vaccine locations should have the necessary supplies and trained staff members available to manage anaphylaxis, implement postvaccination observation periods, immediately treat persons experiencing anaphylaxis signs and symptoms with intramuscular injection of epinephrine, and transport patients to facilities where they can receive advanced medical care. In addition, all patients should be instructed to seek immediate medical care if they develop signs or symptoms of an allergic reaction after their observation period ends and they have left the vaccination location. Health care providers can play an important role in vaccine safety monitoring by being vigilant in recognizing and reporting adverse events after immunization to VAERS at https://vaers.hhs.gov/reportevent.html.

**Summary**

**What is already known about this topic?**

Anaphylaxis is a severe, life-threatening allergic reaction that occurs rarely after vaccination.

**What is added by this report?**

During December 21, 2020–January 10, 2021, monitoring by the Vaccine Adverse Event Reporting System detected 10 cases of anaphylaxis after administration of a reported 4,041,396 first doses of Moderna COVID-19 vaccine (2.5 cases per million doses administered). In nine cases, onset occurred within 15 minutes of vaccination. No anaphylaxis-related deaths were reported.

**What are the implications for public health practice?**

Locations administering COVID-19 vaccines should adhere to CDC guidance, including screening recipients for contraindications and precautions, having necessary supplies and staff members available to manage anaphylaxis, implementing recommended postvaccination observation periods, and immediately treating suspected anaphylaxis with intramuscular epinephrine injection.

## Footnotes

* Brighton level 1 represents the highest level of diagnostic certainty that a reported case is indeed a case of anaphylaxis; levels 2 and 3 represent successively lower levels of diagnostic certainty. Level 4 is a case reported as “anaphylaxis” but that does not meet the Brighton Collaboration case definition. Level 5 is a case that was neither reported as anaphylaxis nor meets the case definition.

† Anaphylaxis and nonanaphylaxis allergic reaction cases with symptom onset occurring later than the day after vaccination (i.e., outside of the 0–1-day risk window) were excluded because of the difficulty in clearly attributing allergic reactions with onset outside this risk window to vaccination.

§ Four of the initial 47 nonanaphylaxis allergic reactions were excluded from the final analysis. Based on the Code of Federal Regulations, a serious adverse event is defined if one of the following is reported: death, life-threatening illness, hospitalization or prolongation of hospitalization, permanent disability, congenital anomaly, or birth defect. https://www.accessdata.fda.gov/scripts/cdrh/cfdocs/cfcfr/cfrsearch.cfm?fr=312.32

¶
https://www.aaaai.org/about-aaaai/newsroom/allergy-statistics
